# Management of Fibromyalgia: An Update

**DOI:** 10.3390/biomedicines12061266

**Published:** 2024-06-06

**Authors:** Eric A. Jones, Farrah Asaad, Nishil Patel, Esha Jain, Alaa Abd-Elsayed

**Affiliations:** 1 Department of Rehabilitation and Human Performance, Mount Sinai Hospital, Icahn School of Medicine at Mount Sinai, New York, NY 10029, USA; farrah.asaad@gmail.com (F.A.); nishilp1010@gmail.com (N.P.); esha.jain346@gmail.com (E.J.); 2 Department of Anesthesiology, University of Wisconsin School of Medicine and Public Health, Madison, WI 53705, USA

**Keywords:** fibromyalgia, pharmacologic management, nonpharmacologic management, antidepressants

## Abstract

Fibromyalgia, a chronic pain condition marked by abnormal pain processing, impacts a significant part of the population, leading to reduced quality of life and function. Hallmark symptoms include widespread persistent pain, sleep disturbances, fatigue, cognitive dysfunction, and mood changes. Through this updated review, we aim to contribute to the evolving understanding and management of fibromyalgia, offering insights into the diverse tools available to improve the lives of those affected by this challenging condition. Management begins with educating patients to ultimately relieve them of unnecessary testing and provide reassurance. Treatment emphasizes a comprehensive approach, combining nonpharmacological interventions such as aforementioned education, exercise, and psychotherapy, alongside pharmacologic management—namely duloxetine, milnacipran, pregabalin, and amitriptyline—which have consistent benefits for a range of symptoms across the spectrum of fibromyalgia. Notably, drugs like nonsteroidal anti-inflammatory drugs (NSAIDs) and acetaminophen are generally not recommended due to limited efficacy and associated risks. Lastly, a variety of other medications have shown promise, including NMDA-receptor antagonists, naltrexone, and cannabinoids; however, they should be used with caution due to a small amount of evidence and potential for adverse effects.

## 1. Introduction

**Figure 1:**
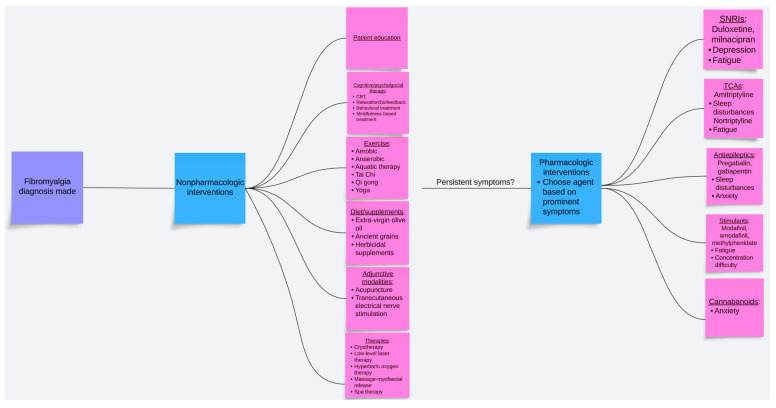
Flow diagram illustrating the multifaceted treatment of fibromyalgia.

Fibromyalgia is a prevalent pain disorder affecting 1–5% of the population, characterized by chronic generalized pain and accompanied by various somatic and psychological symptoms, such as fatigue, sleep disturbances, and anxiety [1,2]. The pathophysiological mechanism remains uncertain, though it is multifactorial, including abnormal pain signaling, genetic predispositions, abnormal neuroendocrine and autonomic system activity, environmental triggers, and sleep disturbances [3]. While efforts to develop nonpharmacologic strategies and pharmacological solutions for fibromyalgia have resulted in three FDA-approved medications, existing data and clinical experiences suggest modest success rates due to a lack of efficacy or side effects, leading to low patient compliance. Current guidelines recommend a multidisciplinary approach that includes cognitive behavioral therapy, exercise, and meditative therapies, alongside medication. The challenges in treating fibromyalgia underscore the complexity of chronic-pain mechanisms. This update, with evidence as recent as February 2024, serves to provide a current clinical guide and summary regarding both the pharmacologic and nonpharmacologic management of fibromyalgia (Table 1, Figure 1).

## 2. Management

### 2.1. Nonpharmacologic Management

#### 2.1.1. Patient Education

Patient education is recommended as the first step in the management of fibromyalgia. It is defined as a set of educational activities planned by professionals that aim to improve a patient’s health by restructuring a patient’s perception of a disease. Patient education, in this way, can reassure a patient in its severity, prognosis, and legitimacy [4].

Education can include varying content focused on pain and stress management or basic knowledge of the disease and its symptoms. Musekamp et al. performed an RCT to compare the effectiveness of patient education to usual education in inpatient rehabilitation. They found that a multidisciplinary treatment program focusing on primary outcome, disease, and treatment knowledge was superior in regard to short-term outcomes and patient satisfaction [5].

Patient education can be beneficial, as it can increase the patient’s adherence to other forms of treatment. It can be in the form of written instruction that gives the patient information on planned treatment strategies and expected results. Additionally, it allows the patient to be a part of his/her treatment plan. Garcia-Rios et al. performed a systematic review to evaluate the effectiveness of patient education for symptoms of fibromyalgia. The authors found 12 articles which showed a significant reduction in perception or understanding, catastrophization, pain intensity, and anxiety of the disease. Therefore, although limited, patient education can serve as a strong first step in the management of fibromyalgia [4].

#### 2.1.2. Cognitive/Psychological Therapy

Psychological therapies have proven effective in other pain disorders, such as chronic pain and chronic lower back pain [8,10]. Some reports have even shown that fibromyalgia patients prefer psychological treatment [9]. Common psychological therapies include cognitive behavioral therapy (CBT), education, relaxation/biofeedback, behavioral treatment, and mindfulness-based treatment. These methods of treatment are designed to alter the psychological principles that underlie the perception of pain, distress, and/or disability. These therapies are often prescribed when treatment shifts from removing the offending agent causing pain to managing pain and the myriad of symptoms affecting quality of life. CBT aims to critically assess repeated thoughts associated with pain, avoidance of painful experiences, and unpleasant thoughts and the resulting consequences of these thoughts. Behavioral therapy targets strongly held beliefs about which behaviors worsen or improve pain. Therapy focuses on instead developing behavior orientated towards goal achievement, as it relates to the values of the individual’s quality of life. Education therapy involves informing individuals of central tenets of their treatment programs, including physical and occupational therapy, for example [7].

CBT is largely thought of as the most effective psychological therapy in fibromyalgia. A 2010 meta-analysis showed that CBT was superior to all other psychological treatments in short-term fibromyalgia pain intensity reduction [11]. CBT and relaxation/biofeedback are most effective in reducing sleep problems in people with fibromyalgia, and all psychological treatments prove equally effective in reducing depression. A dose–reduction relationship has also been shown, with greater doses of psychological therapy showing greater reduction in pain intensity and depression scores [11]. A recent 2020 study revealed significant improvement in time in bed, total sleep time, self-perceived sleep quality, greater time in Stage 4 sleep, and reduced amount of light sleep for people with fibromyalgia treated with CBT [6]. In summary, psychological therapy provides an adjunctive effect in the treatment of patients with fibromyalgia, particularly in the surrounding milieu of associated problems, such as sleep, depression, anxiety, and chronic pain.

#### 2.1.3. Exercise

Exercise is becoming an essential part of the management of pain and functional impairment associated with fibromyalgia. Since fibromyalgia does not present with specific pain patterns and no definitive mechanism, it is difficult to offer specific exercise interventions. However, exercise-induced hypoalgesia (EIH) is a proposed mechanism by which a reduction in pain can be achieved through the performance of aerobic exercise. There are several theories behind this that involve a combination of improved muscle oxygenation, endogenous release of opioids via muscle contractions, reduced hyperactivity of the sympathetic system, and improvement in psychosociological factors [12].

Moderate-quality evidence (based upon 2400 patients throughout 34 trials and 47 various exercise interventions) has demonstrated that aerobic exercise can decrease pain intensity, enhance physical function, and reduce the severity of fatigue. However, long-term effects have not yet demonstrated equal or greater benefit [19].

Anaerobic exercise, which can include weight training, sprinting, and plyometrics, is not as commonly used in patients with fibromyalgia. Studies have shown that this patient population reaches the anaerobic threshold much more quickly, meaning that they would reach a level of oxygen consumption much more rapidly, leading to lactic acid buildup. Therefore, not many studies have been performed to observe the effects of anaerobic exercises in fibromyalgia patients [25].

Aquatic therapy is another beneficial exercise shown to reduce pain and fatigue patients with fibromyalgia [18]. A meta-analysis has been shown to decrease pain, demonstrated by improvements in the Visual Analog Score (VAS) and Fibromyalgia Impact Questionnaire (FIQ) scores. Specifically, aquatic physiotherapy in a heated pool can reduce pain and improve function in affected women with fibromyalgia. The working theory is that the heated pool reduces bone overload due to absent concentric forces on muscles [13].

Qi Gong therapy is an alternative form of exercise that is defined by meditative movements, with a strong emphasis on the mind–body connection. Four RCTs have shown improvement in pain, sleep, and overall function in fibromyalgia patients who consistently practiced Qi Gong therapy for 6–8 weeks [20].

Per randomized control trials described by Wang et al., Tai Chi classes administered twice weekly over 12 weeks demonstrated improvement in FIQ and Short-Form 36 (SF-36) health survey questionnaires. Similar trials with weekly 120-min yoga sessions, in addition to performing at-home sessions 5–7 times per week for 8 months, showed improvement in FIQ and SF-36. These studies demonstrated potential benefits of exercise programs that involve a holistic approach to improving the mind–body interrelationship [22].

#### 2.1.4. Diet/Supplements

Alternative nonpharmacological modalities of treatment for fibromyalgia are also being actively studied and integrated into clinical practice. There have been extensive studies on the optimal diet for patients with fibromyalgia to help alleviate systemic inflammation that may be associated with their pain. Studies have shown that extra-virgin olive oil (EVOO) instead of refined olive oil statistically improved FIQ and mental health status in 23 females after 3 weeks likely due to the antioxidant properties from the high concentration of phenolic compounds. Ancient grains, i.e., Khorasan wheat, which contains elevated levels of magnesium, phosphorus, potassium, selenium, and zinc, helped reduce pain, fatigue, and day-time sleepiness. A 4-week intervention in 38 women demonstrated decreased gastrointestinal and fibromyalgia-related symptoms. Overall, following a healthy dietary model with increased consumption of vegetables, EVOO, and foods high in antioxidants has shown positive effects in patients with fibromyalgia [16].

Supplementation use for the treatment of fibromyalgia symptoms has been shown to have some improvement in pain. Supplements such as Chlorella green algae, coenzyme Q10, acetyl-L-carnitine, freeze-dried aloe vera gel, and a combination of vitamin C and E have been shown to lead to a significant improvement in pain. However, the studies performed for these supplements have not had large sample sizes and are of poor quality; therefore, further research is needed to assess the effectiveness in treating fibromyalgia [26,27].

#### 2.1.5. Adjunct Modalities

In regard to acupuncture, one study included 50 participants, with 25 participants in the acupuncture group. The largest difference in FIQ scores was seen at 1 month, with the greatest improvement being in fatigue and anxiety [24]. Transcutaneous electrical nerve stimulation has pointed to improvement in pain symptoms; however, further clinical studies for daily use are needed [15].

#### 2.1.6. Therapies

Low-level laser therapy (LLLT) is a noninvasive treatment involving a low emission of light to induce cellular changes that theoretically reduces the production of reactive oxygen species. A meta-analysis of 9 RCTs demonstrated LLLT improved FIQ and pain severity, while reducing the number of tender points. Combined with exercise therapy, both proved more effective than just exercise alone [53]. Theories involve anti-inflammatory effects, endorphin production, and increased pain threshold.

Cryotherapy has shown promise, with existing studies suggesting both local and whole-body cryotherapy provide pain relief and improvement in VAS scores. Studies have shown improvement with whole-body cryotherapy at temperatures of −196 °C, −140 °C, and −110 °C. Regarding local cryotherapy, it has been shown that pain relief lasts for up to twenty-four hours after cold-pack application for only ten minutes [28].

Massage–myofascial release treatment is an effective and noninvasive way to improve multiple dimensions of health in fibromyalgia patients. A randomized control trial was performed with 74 patients which demonstrated significant improvement in pain via the VAS scale; in physical function, as shown by the SF-36; and in anxiety 20 weeks post-intervention. Myofascial release is meant to target dense areas of tissue that are painful and hypomobile, ultimately allowing for muscle relaxation and improved circulation [23].

Spa therapy involves a combination of different modalities that include but are not limited to hydrothermal treatment, physiotherapy, contrast therapy with warm and cold bodies of water, and education. A randomized control trial performed in France followed participants in five different spa therapies throughout one year. There was a significant difference in the FIQ score between the control group who received spa therapy after 6 months and the intervention group that received it immediately. The score was significantly higher up to 12 months. No major improvements were seen with sleep or physical activity. In summary, spa therapy is a safe, effective, and minimally invasive approach that can provide benefit in fibromyalgia patients [14].

Hyperbaric oxygen therapy (HBOT) is a treatment that is actively being studied for its effects on inducing neuroplasticity and improving brain function, while also reducing pain via a decrease in glial cell inflammatory mediators. HBOT utilizes increased pressure to increase plasma oxygen uptake. In a prospective crossover study, female patients with fibromyalgia received 2 months of HBOT. There were notable improvements in pain threshold, FIQ score, and SF-36. In addition, neuronal activity measured using SPECT imaging showed a significant increase. Essentially, HBOT helped normalize pain signal responses in the brain. Hyperactive areas decreased, while underactive areas increased. Thus, physiological changes in the brain may have helped improve the pain threshold and response to painful stimuli [21].

### 2.2. Pharmacologic Management

Pharmacologic management of fibromyalgia may be used in conjunction with the management discussed above; however, drug therapy is only used for the management of symptoms. Drug choices should be made to target the most predominant symptoms a patient may be experiencing, such as pain, sleep disturbance, and psychological distress [48]. There are currently three drug therapies approved by the United States (US)’s Federal Drug Administration (FDA): pregabalin, duloxetine, and milnacipran. Notably, the three FDA-approved medications account for approximately 70% of prescribed medications for fibromyalgia [54]. However, other pharmacologic therapies have also been used with varying levels of evidence regarding their efficacy.

#### 2.2.1. Antidepressants

The association of depression alongside fibromyalgia naturally favors the use of antidepressants; however, the effectiveness of antidepressants has been shown to be independent of their effect on depression [39,40]. As stated above, there are currently two FDA-approved antidepressants for the treatment of fibromyalgia: duloxetine, approved in 2008; and milnacipran, approved in 2009. These serotonin and norepinephrine reuptake inhibitors (SNRIs), as well as the tricyclic antidepressant amitriptyline, have demonstrated the most clinical benefit in patients with fibromyalgia [38]. And even though not FDA-approved, amitriptyline is recommended by all various clinical practice guidelines [32,33,36,37]. Though the mechanism of action of antidepressants amongst patients with fibromyalgia has not been systematically studied, there has been evidence for both serotonergic and noradrenergic transmission in fibromyalgia [38]. Thus, it is suggested that the dual effect of blocking the reuptake of serotonin and norepinephrine plays a role in treating pain and other symptoms of fibromyalgia [41].

Recent studies suggest that, for optimal outcomes, the use of antidepressants should be tailored to patient-specific comorbidities and symptoms. A 2022 systematic review compared the use of amitriptyline with current FDA-approved medications (duloxetine, milnacipran, and pregabalin). Amitriptyline had the greatest association with reduced sleep disturbances and fatigue and improved quality of life. Additionally, amitriptyline reduced patient FIQ scores by 30% [29,31,35,42]. Alternatively, duloxetine had the greatest improvement in pain and depression [35]. Milnacipran was less efficacious but showed significant improvement in pain and fatigue [30]. Interestingly, all therapies showed a greater study drop-out rate, except for amitriptyline. Of note, selective serotonergic reuptake inhibitors (SSRIs) have been shown to improve pain, depression, and overall quality of life, but only to a small extent [34].

When comparing tricyclic antidepressants, amitriptyline has been found to be most effective. A study comparing 118 patients with fibromyalgia found an improvement of 36.5% in the amitriptyline group, 26.6% in the nortriptyline group, and 24% in the placebo group. Furthermore, the mean post-treatment FIQ scores were 39.97 in the amitriptyline group, and 48.78 in the nortriptyline group. Additionally, there were nearly double the amount of side effects in the nortriptyline group [55]. If fatigue is a primary component of patients’ fibromyalgia, secondary amines (nortriptyline and desipramine) may be preferred due to their having fewer sedating effects. Tertiary amines (amitriptyline, doxepin, and imipramine) may be favored in these cases due to their sedating profile. However, no study exists directly comparing doxepin or imipramine to amitriptyline.

Thus, when prescribing antidepressants for fibromyalgia, shared decision making amongst patients and clinicians should be utilized, with a focus placed on which symptoms of fibromyalgia should be targeted. Amitriptyline should be preferred for comorbid sleep disturbances, and duloxetine should be preferred for comorbid depression, fatigue, and overall reduction of pain [34]. Additionally, side-effect profiles and starting at low doses should always be taken into consideration when prescribing these medications. Dose-dependent adverse effects (for example, weight gain with tricyclic antidepressants and sexual dysfunction with SSRIs) and contraindications (hepatic disease with SNRIs) have been well studied and should help guide clinician-treatment choice. Cost and availability may also be taken into consideration, as amitriptyline is generally available as a generic, and it is cheaper than duloxetine and milnacipran. Lastly, clinicians should be realistic with patients about the use of antidepressants, as they rarely relieve all symptoms of fibromyalgia and may provide only small symptomatic relief that must be weighed against a medication’s adverse effects.

#### 2.2.2. Antiepileptics

Pregabalin works by selectively binding to the α2δ-1 and α2δ-2 subunits. The α2δ-1 expression is shown to mainly localize with excitatory neurons, whereas the α2δ-2 subunit localizes largely in inhibitory neurons. By binding to these subunits, pregabalin reduces the release of glutamate in the spinal dorsal horn and reduces the activation of the insula and amygdala during emotional processing, playing a role in the experience of pain. In fibromyalgia, pregabalin is thought to reduce pain by preventing sensory propagation of nociception by inhibiting calcium channels and neurotransmitter release. It has also been shown to reduce glutamate and glutamine levels in the posterior insula, interfering with its connectivity [56]. The exact mechanism of action of gabapentin is unknown. It is known to have a high affinity for binding sites throughout the brain to voltage-gated calcium channels, especially α2δ-1, which inhibits the release of excitatory neurotransmitters in the presynaptic area [57].

Antiepileptics were designed to treat seizures, but they have also since been used to treat neuropathic pain and fibromyalgia [43]. In fibromyalgia, it has been shown that the concentrations of glutamate, the pain facilitatory neurotransmitters, and substance P in the central nervous system are elevated. Both pregabalin and gabapentin target these neurotransmitters, which can help modulate pain, promote physiological sleep, and act as an anxiolytic. As mentioned above, pregabalin was FDA-approved to treat fibromyalgia in 2007.

In a systematic review of the antiepileptic medications used for fibromyalgia, only pregabalin was shown to have moderate efficacy. This particular review showed a number needed to treat (NNT) with a range of 4–14 for 50% or more pain reduction. Other antiepileptic medications reviewed, including clonazepam, phenytoin, valproic acid, carbamazepine, lamotrigine, oxcarbazepine, and topiramate, have little-to-no evidence for pain reduction [43]. 

The adverse effects associated with both pregabalin and gabapentin that have been most commonly reported include dizziness, headache, somnolence, and edema. In pregabalin alone, weight gain is reported as the most common other adverse event [44].

#### 2.2.3. Other

Alternative medications have been studied and evaluated for their use to treat fibromyalgia. These include stimulants, opioids, tramadol, naltrexone, cannabinoids, NMDA antagonists, and sodium oxybate.

The use of stimulants for treatment of fibromyalgia has not been studied greatly; however, one clinical trial has been performed on the use of armodafinil. Armodafinil is the R-enantiomer of racemic modafinil. Both are non-amphetamine stimulants that release dopamine and noradrenaline in the CNS, as well as histamine in the hypothalamus [49]. With these responses, the brain is thought to interpret lower fatigue states in patients. This is used for the treatment of excessive sleepiness associated with both narcolepsy and obstructive sleep apnea. However, for the treatment of fatigue in fibromyalgia, the clinical study showed that there was no significant difference in any effectiveness outcome and failed to treat the fatigue associated with fibromyalgia [46,49,58]. Methylphenidate, a neuronal stimulant that acts by inhibiting dopamine and noradrenaline reuptake, has been shown to enhance mood and concentration in patients with fibromyalgia. However, other symptoms, such as pain, were not reported to improve [49,51].

The use of pure mu-opioid receptor agonists such as codeine, fentanyl, and oxycodone is contraindicated due to poor clinical response and has an increased chance of inducing hyperalgesia [48]. However, tramadol, a weak mu-opioid receptor and reuptake inhibitor of serotonin and norepinephrine has been frequently used for the treatment of fibromyalgia [50]. Low-dose naltrexone has shown beneficial effects when compared against a placebo after 12 weeks of treatment, with a mean difference of 0.34 on a 1–10 pain scale [45].

Cannabinoids have two major active components: tetrahydrocannabinol (THC) and cannabidiol (CBD). THC affects pain due to its psychoactive component. CBD has anti-inflammatory and analgesic traits. The two main cannabinoids, nabilone and dronabinol, have undergone three randomized trials for the treatment of fibromyalgia with conflicting results. The first reported a significant relief of pain after two hours of taking the medication. The second reported not only a reduction in pain but also anxiety and quality of life when compared to placebo. Lastly, the third study found a moderate effect on insomnia when using nabilone compared to amitriptyline; however, it showed no effect on pain or quality of life. Another study found improved pain and quality of life after thirty days of treatment, with no negative impact on financial resources or home environment [59]. Due to these varying results, undergoing more controlled studies is crucial in understanding their role in the management of fibromyalgia.

NMDA receptor antagonists have been shown to play a role in treating fibromyalgia. NMDA receptors are one of three subgroups of glutamate receptors. They are known to be involved in the pathogenesis of central sensitization, for which NMDA antagonists were made as options for treatment of disorders resulting in central sensitization, including fibromyalgia. Two studied NMDA receptor antagonists in fibromyalgia are memantine and ketamine. In a double-blind randomized trial, memantine was found to produce a significant reduction in pain, with a hypothesis that the combination of memantine with pregabalin could achieve therapeutic results in the treatment of fibromyalgia [47]. A systematic review of ketamine for the treatment of fibromyalgia revealed short-term (few hours maximum) pain relief with single, low-dose IV ketamine infusion. Studies involving higher doses of more frequent ketamine infusions are lacking; however, two cases studies suggest greater pain reduction with increased doses, longer duration, and more frequent treatments. Larger prospective placebo-controlled trials are needed to further evaluate the efficacy and safety of ketamine for the treatment of fibromyalgia [60].

Sodium oxybate is thought to reduce non-restorative sleep abnormalities and is currently approved by the FDA for the treatment of narcolepsy. Several randomized trials and controlled trials have been performed and shown to significantly improve the symptoms of fibromyalgia. Similarly for the treatment of narcolepsy, sodium oxybate improved sleep in patients with fibromyalgia, increasing slow-wave sleep duration and delta power and reducing frequent nighttime awakenings. The common adverse effects shown with sodium oxybate are headache, nausea, dizziness, and somnolence. However, due to its concern for abuse, the FDA did not approve sodium oxybate for the management of fibromyalgia [61,62,63].

Notably, the use of NSAIDs is not regarded as useful for the treatment of fibromyalgia. A 2017 Cochrane review showed only a modest amount of very low quality evidence for the use of NSAIDs in fibromyalgia [64]. Furthermore, the use of NSAIDs is not recommended by the European League Against Rheumatism (EULAR) and The Association of the Scientific Medical Societies in Germany (AWMF) [65].

Acetaminophen has been shown to have theoretical benefit for fibromyalgia in in vivo mice models, modulating the endogenous cannabinoid system and serotonin-agonist properties [66,67]. However, studies have revealed limited affect due the proposed lack of effect on central pain processing, a large contributor in fibromyalgia [52].

## 3. Conclusions

The management of fibromyalgia encompasses a multidimensional approach, utilizing both pharmacological and nonpharmacological strategies to address the complex array of symptoms that patients experience.

Nonpharmacologic interventions, such as patient education, psychotherapy, exercise regimens, and dietary modifications, offer promising avenues for alleviating pain and improving overall quality of life. These should be initiated early in the disease course. Regarding psychological therapies, CBT has shown the most efficacy in the reduction of pain intensity, sleep disturbances, and depression among fibromyalgia patients. Exercise, including aerobic exercise, anaerobic exercise, aquatic therapies, Qi Gong therapy, and Tai Chi, has demonstrated positive outcomes in pain reduction and functional improvement, though long-term impacts remain under study. Diet, supplementation, adjunct modalities such as acupuncture and transcutaneous electric stimulation, and various therapies show promise in enhancing management.

Pharmacological treatment remains central to fibromyalgia management. It is widely agreed that the choice of medication should be tailored to the specific symptoms of each patient, with shared decision-making between clinicians and individuals. There are three FDA-approved medications—pregabalin, duloxetine, and milnacipran—and each one shows greater efficacy for particular symptom profiles. Amitriptyline has also shown clinical benefits in fibromyalgia and is considered a first-line agent, particularly for improving sleep and fatigue. While other options, such as tramadol, cannabinoids, and low-dose naltrexone, show promise, caution is advised due to potential adverse effects and limited evidence. The integration of these pharmacological and nonpharmacological approaches is crucial in addressing the multifaceted nature of fibromyalgia, highlighting the need for personalized treatment plans that consider individual symptoms, preferences, and tolerances. 

## Figures and Tables

**Table 1:** Summary of nonpharmacologic and pharmacologic management.

Approaches	Articles	Summary
Patient education	Garcia Rios et al., 2019 [4] Musekamp et al., 2019 [5]	Patient education is defined as a set of educational activities planned by professionals that aims to improve a patient’s health by restructuring a patient’s perception of a disease. It can reassure a patient in its severity, prognosis, and legitimacy.
Cognitive/psychological therapy	Prados et al., 2020 [6] Williams et al., 2020 [7] Eccleston et al., 2009 [8] Jorm et al., 2003 [9] van Tulder et al., 2001 [10] Glombiewski et al., 1995 [11]	Psychological therapies provide an adjunctive effect to the treatment of patients with fibromyalgia, particularly in regard to sleep, depression, anxiety, and chronic pain. The most effective therapy, CBT, aims to critically assess repeated thoughts associated with pain, avoidance of painful experiences, and unpleasant thoughts and the resulting consequences of these thoughts.
Exercise	Neelapala et al., 2023 [12] Galavao-Moreira et al., 2021 [13] Maindet et al., 2021 [14] Dailey et al., 2020 [15] Pagliai et al., 2020 [16] Yeh et al., 2019 [17] Zamuner et al., 2019 [18] Bidonde et al., 2017 [19] Sawynok et al., 2017 [20] Efrati et al., 2015 [21] Busch et al., 2011 [22] Castro-Sanchez et al., 2011 [23] Martin et al., 2006 [24] Lund et al., 2003 [25]	Exercise is an effective part of the management process to help a fibromyalgia patient in various domains. Exercise can be facilitated via means such as Qi Gong therapy, pool therapy, yoga, or Tai Chi. In addition to pain control, it allows for overall better function, reduction in anxiety, and improved sleep, and it has positive benefits on overall health.
Diet/supplements	Lowry et al., 2020 [26] Pagliai et al., 2020 [16] Dykman et al., 1998 [27]	Following a healthy dietary model with increased consumption of vegetables, extra-virgin olive oil, and foods high in antioxidants has shown positive effects in patients with fibromyalgia. Ancient grains and herbicidal supplementation have also shown positive effects; however, further research is needed.
Adjunct modalities	Martin et al., 2006 [24] Dailey et al., 2020 [15]	Acupuncture has shown benefits in fatigue and anxiety, while transcutaneous electrical nerve stimulation has shown improvements in pain symptoms. Limited studies are available, and further research is needed.
Therapies	Maindet et al., 2021 [14] Garcia et al., 2020 [28] Yeh et al., 2019 [17] Efrati et al., 2015 [21] Castro-Sanchez et al., 2011 [23]	Noninvasive therapies provide a diverse range of benefits. LLLT has been shown to improve pain severity and tender points. Cryotherapy provides pain relief up to 24 h through extremely low temperatures. Massage–myofascial release treatment significantly improves pain, physical function, and anxiety, while spa therapy, involving hydrothermal and physiotherapy treatments, offers sustained improvements in FIQ scores. Hyperbaric oxygen therapy (HBOT) is also promising, enhancing brain function and pain thresholds through increased plasma oxygen uptake and neuroplasticity.
Antidepressants	Farag et al., 2022 [29] Calandre et al., 2015 [30] Calandre et al., 2014 [31] Ablin et al., 2013 [32] Fitzcharles et al., 2013 [33] Häuser et al., 2012 [34] Konuk et al., 2010 [35] Carville et al., 2008 [36] Häuser et al., 2008 [37] Moret et al., 2006 [38] Arnold et al., 2005 [39] Briley et al., 2004 [40] Briley et al., 2003 [41] Gür et al., 2002 [42]	Antidepressants are an effective treatment for fibromyalgia, with duloxetine and milnacipran being FDA-approved. The choice of antidepressant should be tailored to individual symptoms. Duloxetine and milnacipran should be used in comorbid depression. Amitriptyline is recommended for sleep disturbances, while nortriptyline has less of a sedating profile and is better suited to treat symptoms of fatigue.
Antiepileptics	Wiffen et al., 2013 [43] Siler et al., 2011 [44]	Antiepileptics such as gabapentin and pregabalin have been shown to modulate pain, promote physiological sleep, and act as an anxiolytic. Pregabalin is one of the three FDA-approved medications for treating fibromyalgia-related symptoms.
Other (Pharmacologic)	Due Bruun et al., 2024 [45] Alorfi et al., 2022 [46] Lowry et al., 2020 [26] Tzadok et al., 2020 [47] Kwiatek et al., 2017 [48] Lawson et al., 2016 [49] Hauser et al., 2014 [50] Katz et al., 2013 [51] Meeus et al., 2013 [52] Dykman et al., 1998 [27]	Other pharmacologic options that have been shown to have benefits for treatment of pain in FM include tramadol, low-dose naltrexone, and NMDA receptor antagonists. Pure mu-opioid agonists have not been shown to have efficacious results, along with NSAIDs and acetaminophen. Additionally, armodafinil and methylphenidate have been shown to improve fatigue, increase concentration, and improve mood in patients with fibromyalgia; however, they require further clinical trials. Lastly, some herbicidal supplements have shown benefits with symptoms related to FM; however, the studies performed have been of poor quality and require further research.

## Data Availability

Not applicable.

## References

[1] Jones G.T., Atzeni F., Beasley M., Flüß E., Sarzi-Puttini P., Macfarlane G.J. (2015). The prevalence of fibromyalgia in the general population: A comparison of the American College of Rheumatology 1990, 2010, and modified 2010 classification criteria. Arthritis Rheumatol..

[2] Smith H.S., Barkin R.L. (2011). Fibromyalgia syndrome: A discussion of the syndrome and pharmacotherapy. Dis. Mon..

[3] Gyorfi M., Rupp A., Abd-Elsayed A. (2022). Fibromyalgia Pathophysiology. Biomedicines.

[4] García-Ríos M.C., Navarro-Ledesma S., Tapia-Haro R.M., Toledano-Moreno S., Casas-Barragán A., Correa-Rodríguez M., Aguilar-Ferrándiz M.E. (2019). Effectiveness of health education in patients with fibromyalgia: A systematic review. Eur. J. Phys. Rehabil. Med..

[5] Musekamp G., Gerlich C., Ehlebracht-König I., Dorn M., Höfter A., Tomiak C., Schlittenhardt D., Faller H., Reusch A. (2019). Evaluation of a self-management patient education programme for fibromyalgia—Results of a cluster-RCT in inpatient rehabilitation. Health Educ. Res..

[6] Prados G., Miro E., Martinez M.P., Sanchez A., Lami M.J., Caliz R. (2020). Combined cognitive-behavioral therapy for fibromyalgia: Effects on polysomnographic parameters and perceived sleep quality. Int. J. Clin. Health Psychol..

[7] Williams A.C.d.C., Fisher E., Hearn L., Eccleston C. (2020). Psychological therapies for the management of chronic pain (excluding headache) in adults. Cochrane Database Syst. Rev..

[8] Eccleston C., Williams A.C.d.C., Morley S. (2009). Psychological therapies for the management of chronic pain (excluding headache) in adults. Cochrane Database Syst. Rev..

[9] Jorm A.F., Griffiths K.M., Christensen H., Korten A.E., Parslow R.A., Rodgers B. (2003). Providing information about the effectiveness of treatment options to depressed people in the community: A randomized controlled trial of effects on mental health literacy, help-seeking and symptoms. Psychol. Med..

[10] van Tulder M.W., Ostelo R., Vlaeyen J.W., Linton S.J., Morley S.J., Assendelft W.J. (2001). Behavioral treatment for chronic low back pain: A systematic review within the framework of the Cochrane Back Review Group. Spine.

[11] Glombiewski J.A., Sawyer A.T., Gutermann J., Koenig K., Rief W., Hofmann S.G. (2010). Psychological treatments for fibromyalgia: A meta-analysis. Pain.

[12] Neelapala Y.V.R., Mercuri D., Macedo L., Hanna S., Kobsar D., Carlesso L. (2023). Mechanisms hypothesized for pain-relieving effects of exercise in fibromyalgia: A scoping review. Ther. Adv. Musculoskelet. Dis..

[13] Galvão-Moreira L.V., de Castro L.O., Moura E.C.R., de Oliveira C.M.B., Neto J.N., Gomes L.M.R.d.S., Leal P.d.C. (2021). Pool-based exercise for amelioration of pain in adults with fibromyalgia syndrome: A systematic review and meta-analysis. Mod. Rheumatol..

[14] Maindet C., Maire A., Vermorel C., Cracowski C., Rolland C., Forestier R., Comte A., Roques C.-F., Serra E., Bosson J.-L. (2021). Spa Therapy for the Treatment of Fibromyalgia: An Open, Randomized Multicenter Trial. J. Pain.

[15] Dailey D.L., Vance C.G.T., Rakel B.A., Zimmerman M.B., Embree J., Merriwether E.N., Geasland K.M., Chimenti R., Williams J.M., Golchha M. (2020). Transcutaneous Electrical Nerve Stimulation Reduces Movement-Evoked Pain and Fatigue: A Randomized, Controlled Trial. Arthritis Rheumatol..

[16] Pagliai G., Giangrandi I., Dinu M., Sofi F., Colombini B. (2020). Nutritional Interventions in the Management of Fibromyalgia Syndrome. Nutrients.

[17] Yeh S.W., Hong C.H., Shih M.C., Tam K.W., Huang Y.H., Kuan Y.C. (2019). Low-Level Laser Therapy for Fibromyalgia: A Systematic Review and Meta-Analysis. Pain Physician.

[18] Zamunér A.R., Andrade C.P., Arca E.A., Avila M.A. (2019). Impact of water therapy on pain management in patients with fibromyalgia: Current perspectives. J. Pain Res..

[19] Bidonde J., Busch A.J., Schachter C.L., Overend T.J., Kim S.Y., Góes S.M., Boden C., Foulds H.J. (2017). Aerobic exercise training for adults with fibromyalgia. Cochrane Database Syst. Rev..

[20] Sawynok J., Lynch M.E. (2017). Qigong and Fibromyalgia circa 2017. Medicines.

[21] Efrati S., Golan H., Bechor Y., Faran Y., Daphna-Tekoah S., Sekler G., Fishlev G., Ablin J.N., Bergan J., Volkov O. (2015). Hyperbaric oxygen therapy can diminish fibromyalgia syndrome—Prospective clinical trial. PLoS ONE.

[22] Busch A.J., Webber S.C., Brachaniec M., Bidonde J., Bello-Haas V.D., Danyliw A.D., Overend T.J., Richards R.S., Sawant A., Schachter C.L. (2011). Exercise therapy for fibromyalgia. Curr. Pain Headache Rep..

[23] Castro-Sánchez A.M., Matarán-Peñarrocha G.A., Granero-Molina J., Aguilera-Manrique G., Quesada-Rubio J.M., Moreno-Lorenzo C. (2011). Benefits of massage-myofascial release therapy on pain, anxiety, quality of sleep, depression, and quality of life in patients with fibromyalgia. Evid. Based Complement. Altern. Med..

[24] Martin D.P., Sletten C.D., Williams B.A., Berger I.H. (2006). Improvement in fibromyalgia symptoms with acupuncture: Results of a randomized controlled trial. Mayo Clin. Proc..

[25] Lund E., Kendall S., Janerot-Sjöberg B., Bengtsson A. (2003). Muscle metabolism in fibromyalgia studied by P-31 magnetic resonance spectroscopy during aerobic and anaerobic exercise. Scand. J. Rheumatol..

[26] Lowry E., Marley J., McVeigh J.G., McSorley E., Allsopp P., Kerr D. (2020). Dietary Interventions in the Management of Fibromyalgia: A Systematic Review and Best-Evidence Synthesis. Nutrients.

[27] Dykman K.D., Tone C., Ford C., Dykman R.A. (1998). The effects of nutritional supplements on the symptoms of fibromyalgia and chronic fatigue syndrome. Integr. Physiol. Behav. Sci..

[28] Garcia C., Karri J., Zacharias N.A., Abd-Elsayed A. (2021). Use of Cryotherapy for Managing Chronic Pain: An Evidence-Based Narrative. Pain Ther..

[29] Farag H.M., Yunusa I., Goswami H., Sultan I., Doucette J.A., Eguale T. (2022). Comparison of Amitriptyline and US Food and Drug Administration-Approved Treatments for Fibromyalgia: A Systematic Review and Network Meta-analysis. JAMA Netw. Open.

[30] Calandre E.P., Rico-Villademoros F., Slim M. (2015). An update on pharmacotherapy for the treatment of fibromyalgia. Expert Opin. Pharmacother..

[31] Calandre E.P., Rico-Villademoros F., Galán J., Molina-Barea R., Vilchez J.S., Rodriguez-Lopez C.M., Hidalgo-Tallon J., Morillas-Arques P. (2014). Quetiapine extended-release (Seroquel-XR) versus amitriptyline monotherapy for treating patients with fibromyalgia: A 16-week, randomized, flexible-dose, open-label trial. Psychopharmacology.

[32] Ablin J., Fitzcharles M.-A., Buskila D., Shir Y., Sommer C., Häuser W. (2013). Treatment of fibromyalgia syndrome: Recommendations of recent evidence-based interdisciplinary guidelines with special emphasis on complementary and alternative therapies. Evid. Based Complement. Altern. Med..

[33] Fitzcharles M.-A., Ste-Marie P.A., Goldenberg D.L., Pereira J.X., Abbey S., Choinière M., Ko G., Moulin D.E., Panopalis P., Proulx J. (2013). 2012 Canadian Guidelines for the diagnosis and management of fibromyalgia syndrome: Executive summary. Pain Res. Manag..

[34] Hauser W., Wolfe F., Tölle T., Üçeyler N., Sommer C. (2012). The role of antidepressants in the management of fibromyalgia syndrome: A systematic review and meta-analysis. CNS Drugs.

[35] Konuk N., Ortancil O., Bostanci B., Kiran S., Sapmaz P. (2010). A Comparison of Reboxetine and Amitriptyline in the Treatment of Fibromyalgia Syndrome with Co-Morbid Depressive Symptoms: An Open-Label Preliminary Study. Klin. Psikofarmakol. Bülteni-Bull. Clin. Psychopharmacol..

[36] Carville S.F., Arendt-Nielsen S., Bliddal H., Blotman F., Branco J.C., Buskila D., Silva J.A.P.D., Danneskiold-Samsøe B., Dincer F., Henriksson C. (2008). EULAR evidence-based recommendations for the management of fibromyalgia syndrome. Ann. Rheum. Dis..

[37] Hauser W., Arnold B., Eich W., Felde E., Flügge C., Henningsen P., Herrmann M., Köllner V., Kühn E., Nutzinger D. (2008). Management of fibromyalgia syndrome—An interdisciplinary evidence-based guideline. GMS Ger. Med. Sci..

[38] Moret C., Briley M. (2006). Antidepressants in the treatment of fibromyalgia. Neuropsychiatr. Dis. Treat..

[39] Arnold L.M., Rosen A., Pritchett Y.L., D’Souza D.N., Goldstein D.J., Iyengar S., Wernicke J.F. (2005). A randomized, double-blind, placebo-controlled trial of duloxetine in the treatment of women with fibromyalgia with or without major depressive disorder. Pain.

[40] Briley M. (2004). Clinical experience with dual action antidepressants in different chronic pain syndromes. Hum. Psychopharmacol. Clin. Exp..

[41] Briley M., Moret C. (2003). Fibromyalgia syndrome: An overview of potential drug targets. Idrugs.

[42] Gür A., Karakoc M., Nas K., Cevik R., Sarac A., Ataoglu S. (2002). Effects of low power laser and low dose amitriptyline therapy on clinical symptoms and quality of life in fibromyalgia: A single-blind, placebo-controlled trial. Rheumatol. Int..

[43] Wiffen P.J., Derry S., Moore R.A., Aldington D., Cole P., Rice A.S., Lunn M.P., Hamunen K., Haanpaa M., Kalso E.A. (2013). Antiepileptic drugs for neuropathic pain and fibromyalgia—An overview of Cochrane reviews. Cochrane Database Syst. Rev..

[44] Siler A.C., Gardner H., Yanit K., Cushman T., McDonagh M. (2011). Systematic review of the comparative effectiveness of antiepileptic drugs for fibromyalgia. J. Pain.

[45] Bruun K.D., Christensen R., Amris K., Vaegter H.B., Blichfeldt-Eckhardt M.R., Bye-Møller L., Holsgaard-Larsen A., Toft P. (2024). Naltrexone 6 mg once daily versus placebo in women with fibromyalgia: A randomised, double-blind, placebo-controlled trial. Lancet Rheumatol..

[46] Alorfi N.M. (2022). Pharmacological treatments of fibromyalgia in adults; overview of phase IV clinical trials. Front. Pharmacol..

[47] Tzadok R., Ablin J.N. (2020). Current and Emerging Pharmacotherapy for Fibromyalgia. Pain Res. Manag..

[48] Kwiatek R. (2017). Treatment of fibromyalgia. Aust. Prescr..

[49] Lawson K. (2016). Potential drug therapies for the treatment of fibromyalgia. Expert Opin. Investig. Drugs.

[50] Häuser W., Walitt B., Fitzcharles M.-A., Sommer C. (2014). Review of pharmacological therapies in fibromyalgia syndrome. Arthritis Res. Ther..

[51] Robert S., Katz H.B., Leavitt F. (2013). Methylphenidate Improves Concentration, Energy and Mood in Fibromyalgia.

[52] Meeus M., Ickmans K., Struyf F., Hermans L., Van Noesel K., Oderkerk J., Declerck L.S., Moorkens G., Hans G., Grosemans S. (2013). Does acetaminophen activate endogenous pain inhibition in chronic fatigue syndrome/fibromyalgia and rheumatoid arthritis? A double-blind randomized controlled cross-over trial. Pain Physician.

[53] Hong W.K., Kim Y.J., Lee Y.R., Jeong H.I., Kim K.H., Ko S.-G. (2023). Effectiveness of electroacupuncture on anxiety: A systematic review and meta-analysis of randomized controlled trials. Front. Psychol..

[54] Assessment of the U.S. Fibromyalgia Market in Support of S1 Filing (Phase 2). www.sec.gov/Archives/edgar/data/1430306/000101376211002697/exh9902.pdf.

[55] Heymann R.E., Helfenstein M., Feldman D. (2001). A double-blind, randomized, controlled study of amitriptyline, nortriptyline and placebo in patients with fibromyalgia. An analysis of outcome measures. Clin. Exp. Rheumatol..

[56] Alles S.R.A., Cain S.M., Snutch T.P. (2020). Pregabalin as a Pain Therapeutic: Beyond Calcium Channels. Front. Cell. Neurosci..

[57] Yasaei R., Katta S., Patel P., Saadabadi A. (2024). Gabapentin. StatPearls.

[58] Schwartz T.L., Siddiqui U.A., Raza S., Morell M. (2010). Armodafinil for fibromyalgia fatigue. Ann. Pharmacother..

[59] Abd-Elsayed A., Gilligan C. (2023). Cannabis for treating fibromyalgia. Pain Pract..

[60] Pastrak M., Abd-Elsayed A., Ma F., Vrooman B., Visnjevac O. (2021). Systematic Review of the Use of Intravenous Ketamine for Fibromyalgia. Ochsner J..

[61] Staud R. (2011). Sodium oxybate for the treatment of fibromyalgia. Expert Opin. Pharmacother..

[62] Russell I.J., Perkins A.T., Michalek J.E. (2008). Oxybate SXB-26 Fibromyalgia Syndrome Study Group Sodium oxybate relieves pain and improves function in fibromyalgia syndrome: A randomized, double-blind, placebo-controlled, multicenter clinical trial. Arthritis Rheum..

[63] Spaeth M., Bennett R.M., Benson B.A., Wang Y.G., Lai C., Choy E.H. (2012). Sodium oxybate therapy provides multidimensional improvement in fibromyalgia: Results of an international phase 3 trial. Ann. Rheum. Dis..

[64] Derry S., Wiffen P.J., Häuser W., Mücke M., Tölle T.R., Bell R.F., Moore R.A. (2017). Oral nonsteroidal anti-inflammatory drugs for fibromyalgia in adults. Cochrane Database Syst. Rev..

[65] Kia S., Choy E. (2017). Update on Treatment Guideline in Fibromyalgia Syndrome with Focus on Pharmacology. Biomedicines.

[66] Högestätt E.D., Jönsson B.A.G., Ermund A., Andersson D.A., Björk H., Alexander J.P., Cravatt B.F., Basbaum A.I., Zygmunt P.M. (2005). Conversion of acetaminophen to the bioactive N-acylphenolamine AM404 via fatty acid amide hydrolase-dependent arachidonic acid conjugation in the nervous system. J. Biol. Chem..

[67] Gould G.G., Seillier A., Weiss G., Giuffrida A., Burke T.F., Hensler J.G., Rock C., Tristan A., McMahon L.R., Salazar A. (2012). Acetaminophen differentially enhances social behavior and cortical cannabinoid levels in inbred mice. Prog. Neuro-Psychopharmacol. Biol. Psychiatry.

